# *Hipparion* tracks and horses' toes: the evolution of the equid single hoof

**DOI:** 10.1098/rsos.230358

**Published:** 2023-06-21

**Authors:** Alan R. Vincelette, Elise Renders, Kathleen M. Scott, Peter L. Falkingham, Christine M. Janis

**Affiliations:** ^1^ Department of Pathology, St. John's Seminary, Camarillo, 93012, CA, USA; ^2^ Department of Functional Morphology, Faculty of Veterinary Medicine, Utrecht University (Ret.), Utrecht, 3584 CM, The Netherlands; ^3^ Department of Cell Biology and Neuroscience, Rutgers University, New Brunswick, 08854, NJ, USA; ^4^ School of Biological and Environmental Sciences, Liverpool John Moores University, Liverpool, L3 3AF, UK; ^5^ Bristol Palaeobiology Group, School of Earth Sciences, University of Bristol, Bristol, BS8 1RJ, UK; ^6^ Department of Ecology and Evolutionary Biology, Brown University, Providence, 02912, RI, USA

**Keywords:** horse, *Equus*, limb evolution, digit reduction, hoof, frog

## Abstract

The traditional story of the evolution of the horse (family Equidae) has been in large part about the evolution of their feet. How did modern horses come to have a single toe (digit III), with the hoof bearing a characteristic V-shaped keratinous frog on the sole, and what happened to the other digits? While it has long been known that the proximal portions of digits II and IV are retained as the splint bones, a recent hypothesis suggested that the distal portion of these digits have also been retained as part of the frog, drawing upon the famous Laetoli footprints of the tridactyl (three-toed) equid *Hipparion* as part of the evidence. We show here that, while there is good anatomical and embryological evidence for the proximal portions of all the accessory digits (i.e. I and V, as well as II and IV) being retained in the feet of modern horses, evidence is lacking for the retention of any distal portions of these digits. There is also good ichnological evidence that many tridactyl equids possessed a frog, and that the frog has been part of the equid foot for much of equid evolutionary history.

## 1. Introduction

**Figure 1.**
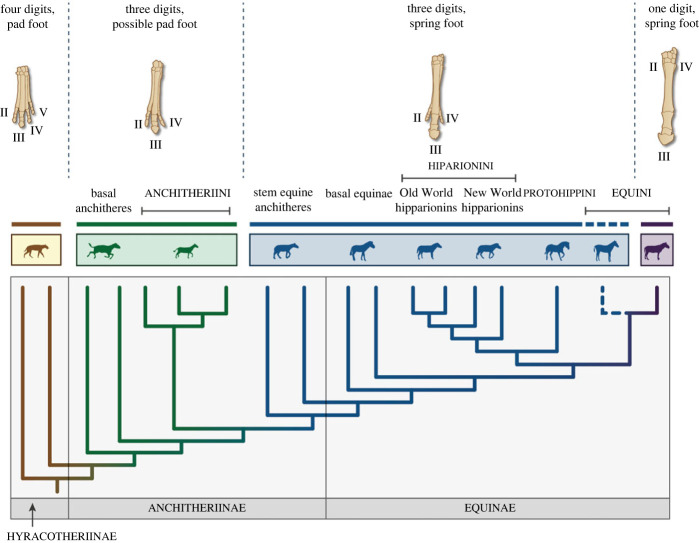
Phylogeny showing equid relationships, plus left front feet of selected taxa showing reduction of toes over time, and the transition from pad foot to spring foot. The hatched blue line represents the condition of side toe reduction (= incipiently monodactyl) in tridactyl species of *Pliohippus*. Pictures of feet from [10], phylogeny modified from [11], figure by Nuria Melisa Morales-García. Credits for silhouettes (all from phylopic.org unless otherwise stated) are as follows, from left to right: *Eohippus angustidens* by Scott Hartman; *Mesohippus* by Heinrich Harder, vectorized by T. Michael Keesey; *Hypohippus* (Wikipedia); *Anchitherium* by Zimices; *Archaeohippus* (Wikipedia); *Merychippus insignis* (this species not actually basal) (Wikipedia); *Merychippus* (standing in for *Protohippus*) by Mercedes Yrayzoz, vectorized by T. Michael Keesey; *Neohipparion affine* by Bruce Robert Horsefall, vectorized by Zimices; *Hippotherium primigenium* by Zimices; *Pliohippus* by Zimices; *Equus scotti* by Bruce Robert Horsefall, vectorized by Zimices. Wikipedia images in the public domain. Phylopic images available for reuse under the Public Domain Dedication 1.0 license, or Creative Commons Attribution unported license http://creativecommons.org/licenses/by/3.0/.

**Figure 2.**
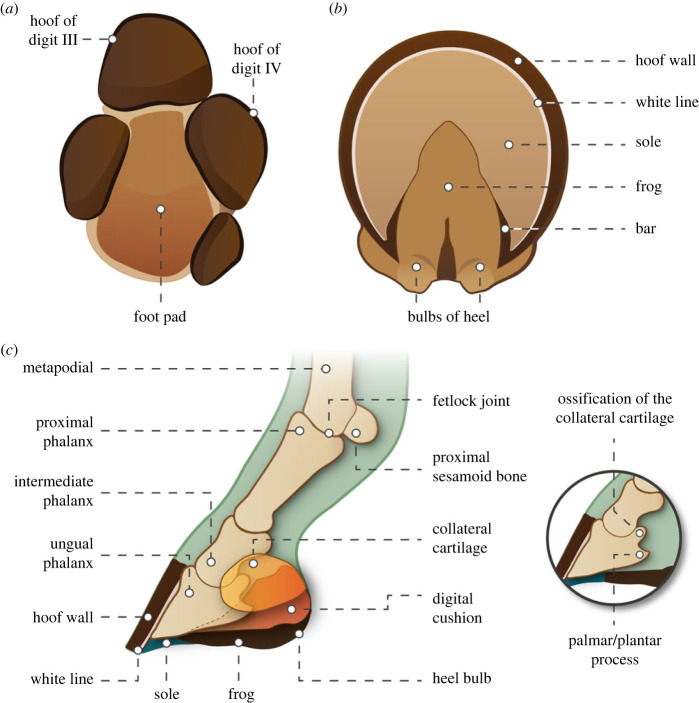
(*a*) Plantar view of padded tetradactyl left fore foot of a tapir (*Tapirus indicus*), probably resembling feet of hyracotherine equids; (*b*) plantar view of hoof of modern horse (*Equus caballus*); (*c*) cut-away diagram of a modern horse foot showing the position and orientation of the ungual phalanx, plus the plantar processes and collateral cartilages. The navicular (distal sesamoid) bone is not shown. Figure by Nuria Melisa Morales-García: (*a*) from photographs supplied by Jamie MacLaren; (*b*) modified from Wikipedia; (*c*) based on a diversity of sources, including figs. 23.21, 22 and 23 in [12], fig. 4 in [13] and fig. 14.8–3 in [14].

One of the familiar stories of horse evolution (family Equidae) is that of digit reduction and loss, often interpreted as an evolutionary progression. Equids, like other perissodactyls, have a mesaxonic foot with the central digit (digit III) being the predominant one. The basal perissodactyl condition, retained today in tapirs, involves four digits in the manus and three in the pes, with small hooves on each digit and a posterior footpad. Rhinos have lost the fourth manual digit, but unlike most other perissodactyl lineages, equids show further digit reduction in evolution, from the three toes (digits II–IV) of earlier equids (four in the manus of the most basal ones, the hyracotheres) to the single main toe of the extant *Equus*, enclosed in a large single hoof [1–9] (see figures 1 and 2): i.e. a change from the tridactyl to the monodactyl condition. This anatomical change has been interpreted as an adaptation for life in open habitats (e.g. [15]), along with the acquisition of hypsodont (high-crowned) cheek teeth for grazing (or incorporating at least some grass in the diet), and larger body size (e.g. [16]).

The actual pattern of equid evolution is much more complicated than the traditional story: monodactyl (single-toed) equids were only a small part of the diverse large-bodied species with high-crowned (hypsodont) cheek teeth that radiated with the spread of grasslands in the Miocene (see [17] for review). Until the Plio-Pleistocene, the great majority of hypsodont equids, in both the New and Old Worlds, were the persistently tridactyl (three-toed) hipparions (subfamilies Protohippini and Hipparionini), often coexisting with monodactyl equin (subfamily Equini) taxa. Despite having three toes rather than one, how similar were the feet of these equids to those of modern horses?

Figure 1 shows a simplified phylogeny of the Equidae and illustrates the different types of feet. A distinguishing feature of the single hoof of extant equids is the V-shaped keratinous frog on the sole (figure 2*b*), a structure not seen in other hoofed mammals such as ruminants that retain two main digits on each foot, and hence have two smaller hooves (i.e. cloven-hoofed). Is the frog unique to monodactyl horses or does it have a longer evolutionary history, originating among multi-toed equids? In this contribution we address the issue of digit loss in equids, the formation of the modern type of equid hoof, and whether any portions of the supposedly lost digits have been retained in the equid hoof, as recently proposed [18].

## 2. Methods

Our methodology mainly applies to the measurements used for the ovality index (OI) described later, i.e. to the maximum length and width of the ungual phalanx of the third digit (*n* = 225) or of the hoof prints (*n* = 44). Measurements of ungual phalanges were taken by C.M.J. with vernier callipers from museum specimens, or calculated by A.R.V. based upon measurements of photographic material in the literature that provided a scale or bone measurement (details provided in electronic supplementary material, table S1). Measurements of hoof prints were taken from photographs with a scale in the literature or from personal direct measurements by A.R.V. or E.R. (details provided in the electronic supplementary material, table S2).

The ungual phalanx III and hoof print OI data were subjected to two-sample Welch's *t*-tests and two-sample Mann–Whitney *U*-tests using the XLSTAT add-in for Excel, to quantitatively test for significant shape differences between tridactyl and monodactyl groups of equids. For the third digit ungual phalanx data, four different analyses were performed: all equids (127 tridactyl, 98 monodactyl); all equids less hyracotheres (i.e. anchitheriines and equines, 110 tridactyl, 98 monodactyl); equines only (65 tridactyl, 98 monodactyl); and equines less *Equus ferus caballus* (65 tridactyl, 78 monodactyl), as the domestic horse (*E. ferus caballus*) has highly varied phalanx shapes. For the hoof print data two different analyses were performed: all anchitheriines and equines (there were no hyracotheriines in this dataset) (24 tridactyl, 22 monodactyl); and equines less South American and hemionine taxa (24 tridactyl, 10 monodactyl). The results, and the justifications for these groupings, are discussed later and displayed in electronic supplementary material, table S3. In addition, photographs were taken with a 12 MP Samsung camera (ƒ/1.6 aperture, 28 mm lens) of the footprints left by tapirs, Icelandic horses, Shetland ponies, Sicilian donkeys and Tennessee walking horses in various substrates (sand, dirt and mud) and in various gaits (walk, trot, tölt and gallop). These are found in figures 6 and 8, electronic supplementary material, figures S4, S5 and S10. In a number of cases several different individuals were measured, especially in the case of extant taxa; the measurements were not averaged.

Height maps (in greyscale and rainbow) were produced by P.L.F. for tapir and modern horse prints (in electronic supplementary material, figure S1) via photogrammetry. Models were produced using Reality Capture and then oriented and coloured using Blender (for more on the methodology see [19]). Finally, photographs were taken with the same camera noted above of a Mangalarga Marchador horse in a marcha picada gait for electronic supplementary material, figure S3.

## 3. The evolutionary history of the *Equus* foot

### 3.1. The evolution of monodactyly and the spring foot

The feet of extant equids are usually considered to be distinguished by the monodactyl condition, but a more important event in equid pedal evolution may have been the acquisition of the ‘spring foot’ in the subfamily Equinae (plus some derived species in the paraphyletic family Anchitheriinae) [20–23] (see [17] for review). This involved the transformation of the padded foot with a subunguligrade foot posture (probably resembling that seen today in rhinos and tapirs, figure 2*a*) found in basal equids (Hyracotheriinae and probably also most Anchitheriinae) to the fully unguligrade foot posture (standing on the ungual phalanx, also known as the third or distal phalanx, or the coffin bone in modern horses), seen in the Equinae and derived Anchitheriinae. The third digit now became even more predominant (as is true for all anchitheres, figure 1) and was probably encased in a keratinous hoof (figure 2*b,c*). In tridactyl spring-footed equids, digits II and IV (medial and lateral to the central digits; also known as the ‘side toes’, referred to here as side digits) were shorter than in more basal species, and most likely bore small hooves. As these toes did not contact the ground during normal locomotion (see later discussion), these equids have been termed ‘functionally monodactyl’ [2,21,24].

**Figure 3.**
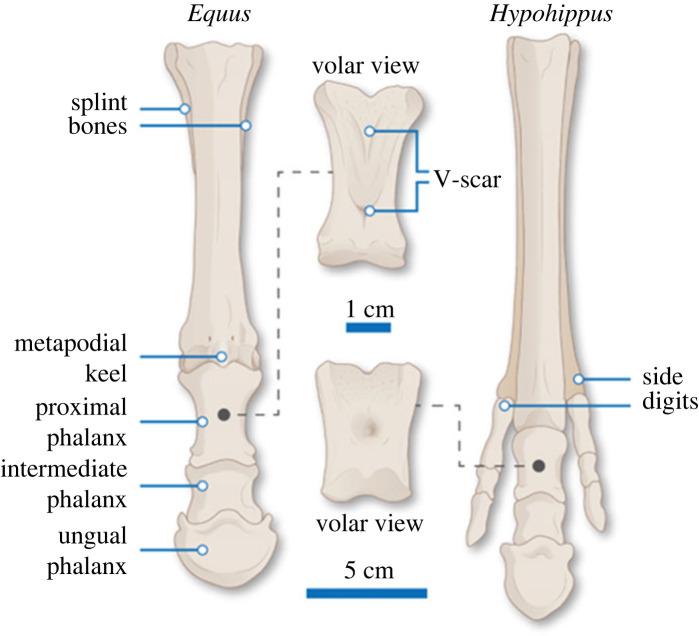
Left manus of a spring-footed monodactyl equid (*Equus burchelli*, based on MCZ 5003) and a tridactyl equid that may have retained a footpad (*Hypohippus equinus,* based on an Alamy stock figure from [29], with some modifications from photographs taken by C.M.J. of *Hypohippus* specimen AM 60545). The 1 cm bar refers to the volar (plantar) view of the proximal phalanges and the 5 cm bar refers to the cranial view of the whole manus. Note in *Equus* the elongated proximal phalanx, elevating the metacarpal-phalangeal (fetlock) joint; the prominent plantar V-scar of this phalanx for the attachment of the oblique suspensory ligaments; the cranial extension of the distal keel on the metacarpal limiting the motion at the fetlock joint to the parasagittal plane. Figure (and original) by Nuria Melisa Morales-García (modified from [30]).

The spring foot functions to enhance the storage of elastic energy during locomotion, thereby increasing locomotor efficiency [25]. The metapodial-phalangeal (fetlock) joints are now supported by a ligamentary suspensory apparatus that allows for extreme extension (= dorsiflexion) of the fetlock during locomotion with reduced risk of joint damage, which in turn maximizes the storage of elastic energy in the flexor tendons (superficial and deep digital flexors) [22,23,26–28]. Although these soft tissues do not preserve, the form of the spring foot is reflected in its bony anatomy (figure 3), which is first seen in derived anchitheres such as *Parahippus* [31] and is present in all members of the Equinae. An understanding of the anatomy of this derived type of equid foot is important for the later discussion of the fate of the ‘lost’ digits in monodactyl equids.

The reason for the evolution of monodactyly in one tribe of the Equinae, the Equini (including extant *Equus* and several extinct genera such as *Pliohippus*, *Dinohippus*, *Astrohippus* and *Hippidion*), has long been proposed as enhancing galloping speed by lightening the distal foot (e.g. [32]), but may be simply for more general locomotor efficiency at slower gaits such as the trot [17]. Meanwhile, the loss of the side digits may not be adaptive *per se*, but rather reflect a strengthening and lengthening of the middle toe. However, the reasons for monodactyly are not at issue here: the question is what happened to the side digits, and do they still form part of the foot in extant horses?

As discussed below, it is not controversial that the proximal portions of the side digits (at least II and IV) are retained, but a novel hypothesis has recently been presented by Solounias *et al*. [18] that the distal portions of all digits in addition to the central one (digit III) are also retained and now form part of the horse's hoof. This hypothesis would necessitate the frog of monodactyl equids being an evolutionary novelty (apomorphy) and the retention of a padded foot in all tridactyl equids.

### 3.2. Loss of digits in equids and possible retention of digital remnants

As has been known for some time [33], extant monodactyl horses retain proximal remnants of the second and fourth metapodials, although the associated phalanges have been entirely lost. These side metapodials are quite robust and form part of the support of the carpus and tarsus. The distal portions taper along the sides of the third metatarsal to approximately a third to a half its length and are colloquially known as ‘splint bones’ (see figures 1 and 3).

Developmental studies [34] show that early *Equus* embryos go through a stage where there are five distinct digit condensations, reflecting the ancient pentadactyl limb pattern. Although there are no obvious remnants of digits I and V in extant *Equus*, many extinct tridactyl forms (including incipiently monodactyl species of *Pliohippus* with greatly reduced side toes) show evidence of small bone nubbins on top of the proximal portions of the second and fourth metapodials that may represent remnants of digits I and V (see [35] their fig. 7; [17] their fig. 5). Thus, there is good evidence, both developmental and osteological, that proximal portions of all five digits are retained in some format in all equids. This supports the insightful conjecture [18] that, although there are no obviously visible remnants of the proximal portions of digits one and five, these are preserved as ridges on the plantar surface of metapodials two and four. Evidence is also provided from carpal articulations for the presence of remnants of these outermost digits in the manus [18].

However, the notion of *Equus* pentadactyly has been taken a step further, with the proposal that, while the central elements of all the additional digits are absent, remnants of the distal portions of digits I, II, IV and V are preserved within the hoof of the third digit [18]. It is also claimed [18] that the distal portions of digits I and V are retained as the ‘hoof wings’ of the ungual phalanx of digit III (but see discussion in §4.3), while the distal portions of digits II and IV are expressed in the largely keratinous V-shaped ‘frog’ (figure 2*b–c*) on the plantar surface of the hoof (fig. 11 in [18]). An argument is also presented [18] that the so-called keratinous ‘claw’ seen on the front of the hoof in some equid fetuses was retained in the adults of tridactyl equids and is reflected in the cleft in the anterior portion of the ungual phalanx of those forms.

To support the proposed retention of distal elements of the ‘extra’ digits, appeal is made [18] to hoof prints of the extinct tridactyl equid *Hipparion*, and from the anatomy and histology of extant *Equus*, including patterns of foot innervation and arterial supply. We address these matters below.

## 4. Fossil equid trackways and evidence of a frog in tridactyl forms

### 4.1. Side digits, footprints and the frog

Solounias *et al*. [18] reference the trackway made by the tridactyl *Hipparion* sp. (now assigned to *Eurygnathohippus*) from the site of Laetoli (Pliocene of Tanzania, 3.66 Ma) that also contains footprints of the hominin *Australopithecus.* They [18] claim that the footprints show the lack of a frog, supporting their hypothesis that the *Equus* frog is formed from the distal portions of phalanges II and IV, which were still present as complete structures in hipparionin equids. They [18] also conclude that the footprints depicted in [36,37] show that *Hipparion* retained a tapir-like footpad (see figure 2*a*; electronic supplementary material, figure S1*a*). Finally, they consider that the side hoof impressions noted by [37] were not made by digits II and IV, but were instead made by the ‘wings’ of the central hoof, structures that, as noted above, they hypothesize to be remnants of distal portions of digits I and V. We here dispute these interpretations of anatomical and ichnological evidence for the retention of distal remnants of the ‘extra’ digits in the hooves of modern equids.

Firstly, we argue that the apparent side hoof impressions in the Laetoli equid trackway do indeed represent digits II and IV, and we provide evidence of additional trackways of tridactyl equids (hipparions) that also display side hoof impressions. The presence of such impressions implies that these digits were functional and important in equid locomotion, not merely non-functional vestiges that could easily be redeployed as a frog. Second, we question whether it would be possible for the ‘hoof wings’ to leave imprints in the trackways. Finally, we address the issue of whether the frog is a feature unique to monodactyl equids, or perhaps just *Equus*, and provide ichnological evidence that a frog was present in tridactyl equids. This would be consistent with the bony anatomy of the feet of all tridactyl Equinae, which is quite unlike that of the more basal, probably pad-footed equids (such as the anchithere *Mesohippus* discussed in [18]), and shows all the hallmarks of an *Equus*-like spring foot (e.g. a longer proximal phalanx, a distinct V-scar on the plantar side of that phalanx indicative of a suspensory ligament apparatus and a complete distal metapodial keel), including the loss of the footpad (see previous discussion and figure 3).

### 4.2. Laetoli fossil horse footprints

**Figure 4.**
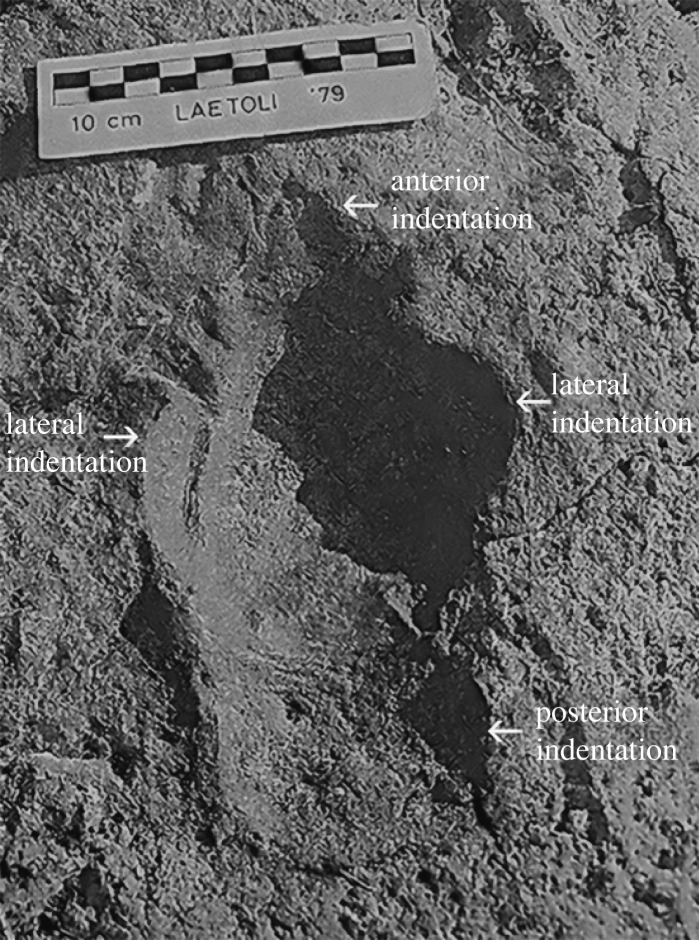
Print 7 of trackway B at Laetoli with medial and lateral impressions located to the side of the central hoof impression (indicated by small arrows), triangular-shaped anterior impression (near ruler) and rectangular-shaped posterior impression (indicated by large arrows) (from [37], p. 474, plate 12.16). Ruler is 10 cm.

**Figure 5.**
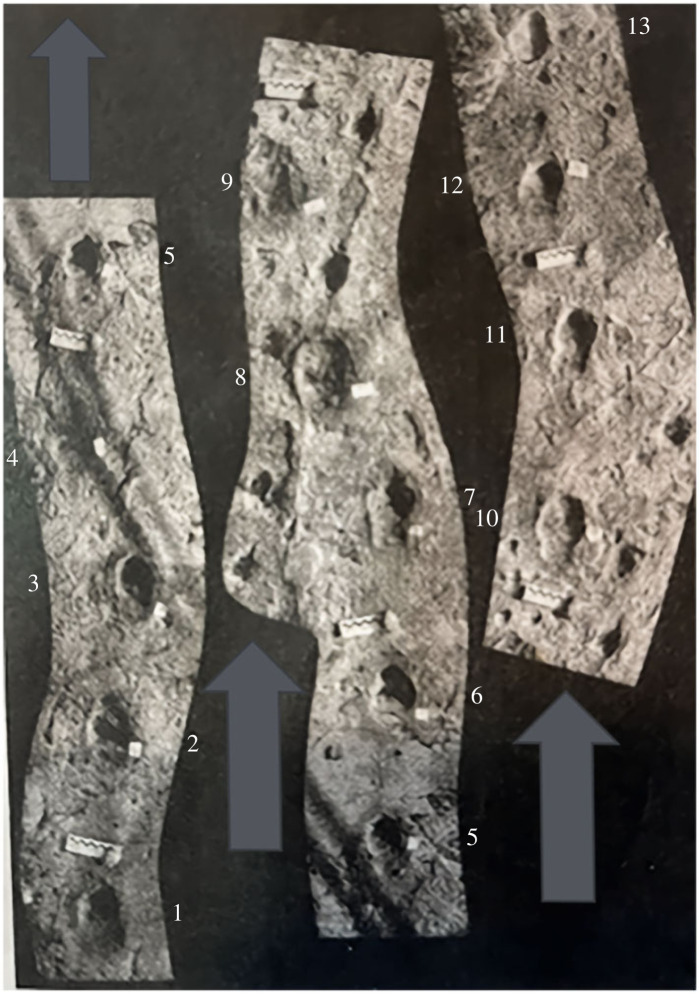
Laetoli equid trackway B with prints (such as B6 and B13) showing more rounded shape anteriorly and regular posterior hoof impressions. Arrows indicate direction of travel (Image from [37], p. 474, fig. 12.18).

Does the Laetoli trackway actually show evidence of imprints from the side digits? Small medial and lateral impressions were first observed in Laetoli *Hipparion* trackway B formed approximately 3.66 Ma (figures 4 and 5) and were interpreted as being made by the hooves of digits II and IV ([36]; [37], p. 476, p. 474, plate 12.16; figure 1). This interpretation has been disputed, with the claim that digits II and IV were so reduced in *Hipparion* that ‘physical constraints might have prevented the side hooves from contacting the mud to create the impression' [18]. It is also postulated [18] that these indentations were instead made by the ‘hoof wings’ of the ungual phalanx of digit III, which were ‘positioned just above the solar surface of the dominant distal phalanx’, and so ‘could plausibly leave impressions in the mud if enveloped in their own keratinous hoof material’. There are, however, several issues with this interpretation, supporting our contention that such impressions were indeed made by digits II and IV.

Biomechanical studies have shown how the side toes of tridactyl equids could have been beneficial by assisting in load bearing, joint stabilization or change of direction [17,21–24,32,37,38]. Most tridactyl Equinae possessed side digits 80% (typically varying between 75% and 85%) the length of the central digit, terminating near the distal end of the proximal phalanx of digit III (figure 1). This proportionality remained quite consistent from 20 to 5 Ma (see [24,39–45]), the exception being tridactyl specimens (some were monodactyl) of *Pliohippus* with greatly reduced side digits (fig. 5*b* in [17]). This consistency in length implies that the side digits retained some sort of functionality.

In extant horses, the proximal phalanges (pastern bones) form a 45–65 degree posterior angle with the ground in a resting position (see electronic supplementary material, figure S2), and the anterior angle between the metapodials and the proximal phalanx of the fetlock joint, which is approximately 165–155 degrees in a resting stance (see electronic supplementary material, figure S2*c*), can reach 120–90 degrees in intermediate-speed to fast gaits [20–23,46–57] (see electronic supplementary material, figure S3). Early tridactyl equids (i.e. basal anchitheres such as *Mesohippus*) had phalanges that formed a 40–45 degree posterior angle with the ground, though with less overall flexibility in the fetlock joint, which had an anterior resting angle of approximately 125 degrees and an angle of approximately 110 degrees at maximum dorsiflexion in fast gaits (see electronic supplementary material, figure S2a: [22,23]). Later tridactyl equids (i.e. Equinae), however, probably had a fetlock joint with a flexibility approaching that of modern horses as well as phalanges forming a posterior angle of approximately 45–55 degrees with the ground, and a fetlock joint that had an anterior resting angle of approximately 130 degrees at rest, achieving an angle of 105 degrees at maximum dorsiflexion (see electronic supplementary material, figure S2*b,c*: favouring the view of [22,23] over that of [21] and [24]). Hence although the side digits of tridactyl spring-footed equines probably did not contact the ground during standard, slow locomotion over firm substrates, they were positioned close enough to the ground (often approximately 5–6 cm above it) to make contact during locomotion in softer substrates, or in faster gaits with forceful impacts. In the former case, the side digits might have provided additional traction or proprioception in order to help avoid slippage and loss of balance, and in the latter case, the side digits could have helped to stabilize the fetlock joint or to provide additional traction useful for quick lateral movements [21,24,37].

Solounias *et al*. [18] also advanced the view that the posterior portion of the Laetoli hoof print B7 (figure 4) represents a tapir-like footpad (their ‘posterior paw’): this would imply that only monodactyl equids had hooves possessing a frog and the type of spring foot seen in extant equids. This type of posterior footprint extension is only seen at Laetoli, and not in other tridactyl footprints of North America and Eurasia, and may relate to the particular substrate of Laetoli (soft ash). One of us [37] had originally interpreted the impression on the posterior end of the Laetoli horse footprints as due to distal mud adhesion; this type of posterior impression can be seen in unshod modern horses when mud fills up the grooves and clumps to the posterior underside of the hoof (see electronic supplementary material, figure S4*a*). Such distal mud adhesions often leave an imprint behind the hoof with an irregular posterior border (see electronic supplementary material, figure S4*b–d*). Another hypothesis is that the posterior impressions were made by the interphalangeal area of the equid limb during extreme dorsiflexion of the fetlock joint at mid-stance in fast gaits (see electronic supplementary material, figure S3, showing such a mid-stance position in modern horses where the proximal phalanx is nearly parallel to the ground).

We think it most likely that these posterior impressions represent an angled entry channel into a soft moist substrate, where the hoof continues in forward motion as it sinks during the early stance phase (subphases 1–2 of the stance according to [58], resulting in a posterior elongation of the print (see [59,60]). Foot impressions made on such a substrate are unlikely to closely record the outline of the foot, but instead often elongate posteriorly as the foot moves forward while it sinks before contacting a hard surface (see electronic supplementary material, figure S5). This elongation may be exaggerated in horses, since horse hooves come down at a slight upward angle with the posterior part (heel) making contact first, and indeed this angle is greater in hind than in front hooves [47,53,61]. Notably, in Laetoli trackway B (figure 5), long posterior impressions are particularly common in hind hoof (pes) prints (B3, B5, B7 and B11) and show up as shorter in front hoof (manus) prints (B2, B8, B10 and B12). Recently discovered equid hoof prints at Laetoli, however, are more regular in appearance and lack the posterior impression (fig. 2 in [62]). Hominin tracks from Laetoli also can possess such posterior extensions, which is usually attributed to the heel being dragged across the substrate before being firmly planted ([62] their figs. 2, 7 and 11, [63]; see electronic supplementary material, figure S6). Hence the posterior extensions of the horse tracks are probably also due to the heel of the hoof dragging, sliding or sinking in the soft deep ash at Laetoli while it moves forward until contacting a hard surface (with perhaps some distal adhesion of sediment). In any case, we dispute that there is an impression of a posterior footpad in Laetoli equids prints, especially as the anatomy of hipparionin foot bones bears the hallmarks of an *Equus*-type of spring foot (figure 3).

Footprints showing a definitive footpad are known from a middle Eocene equid from Utah, USA (probably the hyracothere *Orohippus*, or possibly a small tapiroid such as *Helaletes*) ([64] plate 178, [65]; see also [66]; see electronic supplementary material, figure S7). The tridactyl prints show individual digital pads and a distinct central metapodial footpad, rather like the condition in an extant tapir (figure 2*a*). It is difficult to envisage what a footpad might look like in an equid that had reduced side toes and an enlarged central toe, as in *Hipparion*. An extensive pad projecting behind the ungual phalanx has been envisaged ([18], see their fig. 9*f*), which would account for the length of the footprint impression; but this would require the retention of a tapir-like metapodial pad that seems unlikely with the side digits being so reduced. By contrast, a digital pad of the main digit (as in the two main digits of a tetradactyl pig with reduced side digits, fig. 10*c* in [12]) would be much smaller in extent, and so could not account for the full extent of the Laetoli footprint.

Finally, it was proposed [18] that the somewhat pointed anterior portion of the Laetoli footprint impressions were formed by a midline claw attached to the median cleft of ungual phalanx III, a retention of a feature they claim occurs in neonatal horses. The hipparionin ungual phalanx has an anterior median cleft, not seen in *Equus,* and the phalanx is pointed rather than rounded (fig. 9*e* in [18], and their fig. 9*f* which illustrates the supposed claw on *Hipparion*). A similar ungual phalangeal anatomy can be seen in most tridactyl equids, and in more basal tridactyl or monodactyl Equini such as *Pliohippus pernix* (e.g. F:AM 60803) and *Astrohippus stocki* (e.g. AMNH unnumbered, field number CLAR-400-8295). However, in the somewhat more derived *Dinohippus* (e.g. F:AM 87203) the ungual phalanx is rounded and lacks an anterior cleft, resembling that of *Equus*. The large anchitheriins such as *Hypohippus* (e.g. F:AM 60606) and *Megahippus* (e.g. USNM 175375) also have more rounded ungual phalanges and lack this cleft, and so the presence or absence of a cleft is probably simply related to phalanx shape (see later discussion). The issue of a claw in any equid, neonatal or adult, is further discussed in §5.2 below.

Moreover, other footprints at Laetoli show a more rounded hoof impression anteriorly as in *Equus* (figure 5). Based on its shallowing upwards, we believe that the anterior portion of Laetoli impression B7 probably represents a toe exit trace (as with [59] their fig. 8*a*, impression CBR46.1), rather than the presence of an anterior ‘claw’, that would be expected to exert higher pressures as the foot toes off, resulting in deeper portions of the track. The issue of the possible retention of a fetal claw is discussed further in §5.2.

### 4.3. The position of the ‘hoof wings’ in relation to equid trackways

The medial and lateral palmar and plantar processes (from here on simply termed ‘plantar processes’) of the ungual phalanx support collateral cartilages (also known as ungular cartilages; figure 2*c*) that may act to transfer force of impact to the digital cushion (or subcutaneous tissues) and/or as mechanisms of venous return [13]. These combined structures are termed ‘hoof wings’ in [18]. Contrary to the assertion in [18], the cartilages are not surrounded by their own keratinous material and lie largely outside of the hoof capsule ([13]; see especially their figs. 422 and 431, which clearly show the relationships of the lateral cartilages). However, there is some uncertainty as to what is actually meant by the term ‘hoof wing’ in [18]. In [18] fig. 8, the collateral cartilage is shown as attaching to both the plantar process (which is called the ‘angle’ in their fig. 5) and to a bony projection dorsal to this, which is termed the ‘wing of distal phalanx’ in [18] (their fig. 5). In [18] (their fig. 9), the collateral cartilage is shown as attaching only to this ‘wing’ in *Equus*, but to the plantar process in *Hipparion*. But this bony ‘wing’ is not a natural part of an *Equus* hoof: rather, it is a pathological condition of ossification of a portion of the lateral cartilage known as sidebone, seen mainly on the lateral side of the front hoof in certain modern domestic horses ([67] their fig. 4) as well as in historic domestic horses ([68] their fig. 10). This pathology may be primarily a feature of older horses related to human use, but it has been observed in at least one fossil *Equus* [69]. Figure 2*c* illustrates a small ossification at this site, while the ‘wing’ depicted in [18] represents a pronounced ossification. Thus, it appears that this feature on the hoof of modern *Equus* that was considered to be homologous with the missing distal portion of digits I and V [18] is a pathology rather than a normal condition.

It might still seem possible that the ‘wings’ shown in *Hipparion* ([18], fig. 9*f*), where the collateral cartilage attaches to the plantar process and extends posteriorly would be capable of making the lateral impressions seen in Laetoli print B7 (figure 4), although the depiction in [18] appears to show the cartilages incorporated within the proposed footpad, in which situation they would be unable to make an independent impression. However, in ([18], fig. 9*f*), the ungual phalanges of *Equus* and *Hipparion* are depicted as placed flat on the surface, which is not how the ungual phalanx is positioned within the hoof capsule (although the phalanx is usually placed flat on the ground in mounted horse skeletons). As is clear in X-rays, the phalanx in *Equus* is at an angle of approximately 30 degrees to the ground (figure 2*c*); it is supported ventrally by the digital cushion, and the frog lies distal to the cushion. Thus, these processes and cartilages are elevated dorsally (as they would be in any unguligrade mammal), away from any possibility of contact with the substrate during normal locomotion.

Although we cannot know for sure if *Hipparion* had the same pedal anatomy as *Equus*, it is highly unlikely that the ungual phalanx was placed flat on the ground as depicted by [18]. In tapirs, where the ungual phalanges are encased in small independent hooves, they do not sit flat on the substrate but are elevated by the footpad so that they are positioned at an angle to the ground (see [70], p. 25, their fig. 3). The same is also true for canid ungual phalanges (see [12], fig. 10.19). We thus dispute the notion that the plantar processes and collateral cartilages in tridactyl equids, whatever their possible homology, could have been responsible for any imprints in trackways. The more likely explanation is that they were formed by *Hipparion* side toes.

### 4.4. Evidence of side toes in other trackways of tridactyl equids

In addition to the Laetoli trackway, distinct, small side hoof indentations can be seen in other tridactyl equid trackways. Though it is true that side hooves do not generally leave impressions in tridactyl equine tracks [71–74], a few trackways are now known that contain them. Such side toe impressions occur in two distinct forms.

Some fossil equid trackways display side toe impressions located to the side of the central toe (see electronic supplementary material, figure S8). This occurs in a few hipparionin trackways of the Miocene and Pliocene. Other hipparionin trackways, however, show side toe impressions that occur well behind and clearly separated from the central hoof (see electronic supplementary material, figure S9), reflecting the loss of the pad foot that freed up the side digits. Similar trackways, with posterior side toe imprints, can be seen in extant reindeer [75]. Further details of such equid trackways can be found in the electronic supplementary material.

Thus, the ichnological evidence supports the hypothesis that digits II and IV were indeed able to make contact with the ground during tridactyl equid locomotion, at least in certain substrates, such as soft ones. This presents three challenges to the interpretation presented in [18]. First, it is likely the medial and lateral indentations of tridactyl equid tracks at Laetoli and elsewhere were indeed formed by the side toes rather than by the plantar processes and collateral cartilages of the central digit ungual phalanx. Second, as later Neogene equid side toes make impressions both to the side of and posterior to the central toe, they appear to be freed from any footpad. Third, it is difficult to imagine how such functional side digits made their way into the central hoof as the V-shaped frog in the transition to monodactyly. Rather, it is quite possible that the frog is simply a highly specialized shock-absorbing structure that arose on the plantar surface of the hoof, probably as a specialized anterior extension of the bulbs of the heel, but, in any case, derived independently of any distal digit remnants.

### 4.5. The frog of the equid hoof

The frog is a wedge-shaped structure comprising of soft, elastic horn that forms part of the plantar surface of the hoof; the remainder of the plantar surface is covered by the sole [12]. It is located medially on the posterior plantar surface of the hoof between the V-shaped bars of the hoof wall (figure 2*b*), and it is continuous posteriorly with the bulbs of the heel, forming their anterior extension [12]. Anteriorly and laterally, it is continuous with the sole. Its apex points forward, and its sides (crura) are separated from the hoof wall by two collateral sulci (grooves) and it also bears a central sulcus. In cross-section, the frog is ‘W’ shaped, allowing it to spread and flatten when weighted, and rebound when the weight is lifted [67]. Although it is stated in [18] that the frog bears two separate portions (hooves) that are remnants of its purported origin from digits II and IV, no such structures are present upon examination of the frog of living horses, nor are they described in the extensive veterinary literature (see, e.g. [12,67,76]), and the keratinous layer is quite uniform in appearance.

The frog is heavily cornified, like the hoof wall and sole, but the structure and properties of the keratinous layers of these parts differ, as pointed out in [18]; however, the differences reflect their different functions, not their ontogenetic history. The keratinized sole is largely protective and is not weight-bearing; consequently, the structure of its keratinous covering differs from that of the weight-bearing wall [12,77]. Although the frog normally contacts the ground during locomotion, most of the weight is borne on the hoof wall and transferred to the ungual phalanx through the dermal papillae. This is reflected in the properties of the cornified layer of the epidermis, with the frog having a higher water content than the hoof wall, making it more elastic in nature [78–80]. The frog can thus change in morphology from being wide and spongy, when habituated to wetter, softer terrain, to being narrow and hard, when habituated to hard, smooth terrain [80,81]. This is in part due to wear and environmental influence, but it may also be an adaptive response to maximize traction for each terrain type.

The frog acts primarily as a shock absorber that transmits forces to the digital cushion located deep in it and helps dissipate force during initial ground contact [82–86]. Secondarily, the frog seems to have a role in enhancing traction by providing additional grip [87,88]. Both functions are enhanced by the elastic and pliable consistency that results from the particular structure of the keratin and is maintained by secretions of glands in the digital cushion which lies deep in it [12]. Numerous studies have described these functional differences within different areas of the hoof [77,89–91].

To better understand the structure of the frog, it is helpful to understand homologies with other structures of the equine hoof. The wall of the hoof corresponds to the nail plate in other mammals [92], but in horses is expanded to wrap around the tip of the digit. The sole, frog and bulbs of the heel are specializations of the epithelium and underlying dermal structures and are the homologous equivalents of the digital pad (tip) of the human or carnivore digit or the pad of a ruminant hoof [12,77,93]. Although the prominent triangular frog of modern horses is unique among living ungulates, the bulbs of the heel (see the *Equus* condition in figure 2*b,c*) commonly extend anteriorly onto the plantar surface of the foot to merge with the sole in other perissodactyls and artiodactyls. The frog and heel bulbs have been envisaged as being homologous with the digital pad of pigs and canines, the footpad of tapirs and rhinos, and the bulb of the finger in humans ([12] their figs. 10-18, 10-19).

In functional terms, ruminant artiodactyls, which independently evolved an unguligrade posture and a type of spring foot [12], are probably the most similar animals to living equids. Ruminants have a foot with two separate hooves; while the metapodials are fused, the phalanges are separate, resulting in the cloven-hoofed condition. While ruminants do not possess a distinct structure homologous with the frog, the bulbs of the heels do extend anteriorly onto the plantar surface of the foot to merge with the sole, with the exception that the heel bulbs do not form a separate structure grossly. As in equids, there is a digital cushion underlying the plantar surface of the foot [12]. The weight in ruminants is primarily borne on the sole rather than the wall of the hoof and is transferred to the ungual phalanx through the digital cushion [12]. A similar pattern of weight distribution would also occur in extant rhinos and tapirs, and presumably in pad-footed equid taxa. This suggests that a morphologically distinct frog serving as a shock absorber may have arisen when the weight distribution on the foot shifted from the entire sole to the wall of the hoof capsule. We suggest that this probably occurred with the development of the spring foot and the resultant more upright posture of the phalanges (fig. 3 in [22]) and lengthening of the proximal phalanx. This interpretation, supported by the ichnological data, would predict the presence of a frog in most if not all spring-footed equines regardless of whether they were tridactyl or monodactyl.

More information about the frog, and its other possible functions, is presented in the electronic supplementary material.

### 4.6. Evidence for the frog in fossil equid trackways

**Figure 6.**
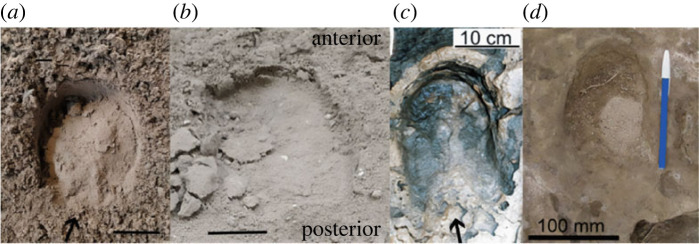
(*a*) Right front hoof print of unshod Tennessee walking horse galloping in dirt showing a triangular frog posteriorly; (*b*) right hind hoof print of unshod Tennessee walking horse galloping in dirt with no noticeable frog posteriorly; (*c*) print PCT-X of *Equus* (*Amerhippus*) or *Hippidion* formed 12 ka at the Pehuen Co site in Argentina with visible triangular frog (from [95, p. 154], their fig. 10D); (*d*) print LDM-S_2_T_4_ of *Equus* (*Amerhippus*) or *Hippidion* from Laguna del Monte, Argentina with hardly any frog visible posteriorly (from [96, p. 233], fig. 9*i*). Bars are 5 cm unless otherwise indicated.

Although the frog sometimes leaves an impression in modern unshod (or shod) horses when the hoof is clean and the substrate apposite [94], the impression left by the frog is most often a subtle triangular outline at the back of the hoof print (figure 6*a*). Moreover, the frog frequently does not leave an impression at all, due to it being filled up or covered over with sediment, or due to collapse of the substrate after the foot is withdrawn (figure 6*b*). Sometimes the print is an outline of the hoof shape alone (as on dry sand or dirt), sometimes just the hoof wall, sole, V-bars and collateral sulci, but not the frog, leave an impression (especially if the frog is full of sediment and the substrate is quite moist), and on occasion the frog itself will make an impression (such as in moist dirt or snow, or a somewhat moist substrate lying on top of a harder one). Additional figures of the footprints of extant horses, showing the presence or absence of a frog impression, can be found in the electronic supplementary material (figures S10 and S1*b*). The same pattern occurs in Pleistocene equid footprints. Occasionally a frog is clearly visible and ‘easily distinguished’ at the back of a footprint (figure 6*c*; see also [97], pp. 313 and 321, figs. 18.1 and 18.11; [98], p. 5, fig. 1; [99], p. 11; [100], pp. 204–205), but often the frog does not show up in the print or is inferred from other features (figure 6*d*; see also [101], p. 217, fig. 14).

Hence, the absence of frog impressions in fossil equid trackways by itself need not indicate the absence of a morphological frog on the hoof. The fact that no frog is present in the Laetoli equid impressions may be more related to substrate and preservation than to the lack of a frog on the hoof. Indeed, the hominid footprints at Laetoli fail to preserve much detail regarding toes or grooves in human feet, and so it is reasonable to assume that the sediment either adhered to the feet, reducing fidelity of tracks, or responded to indentation in such a way as to not preserve subtle detail.

**Figure 7.**
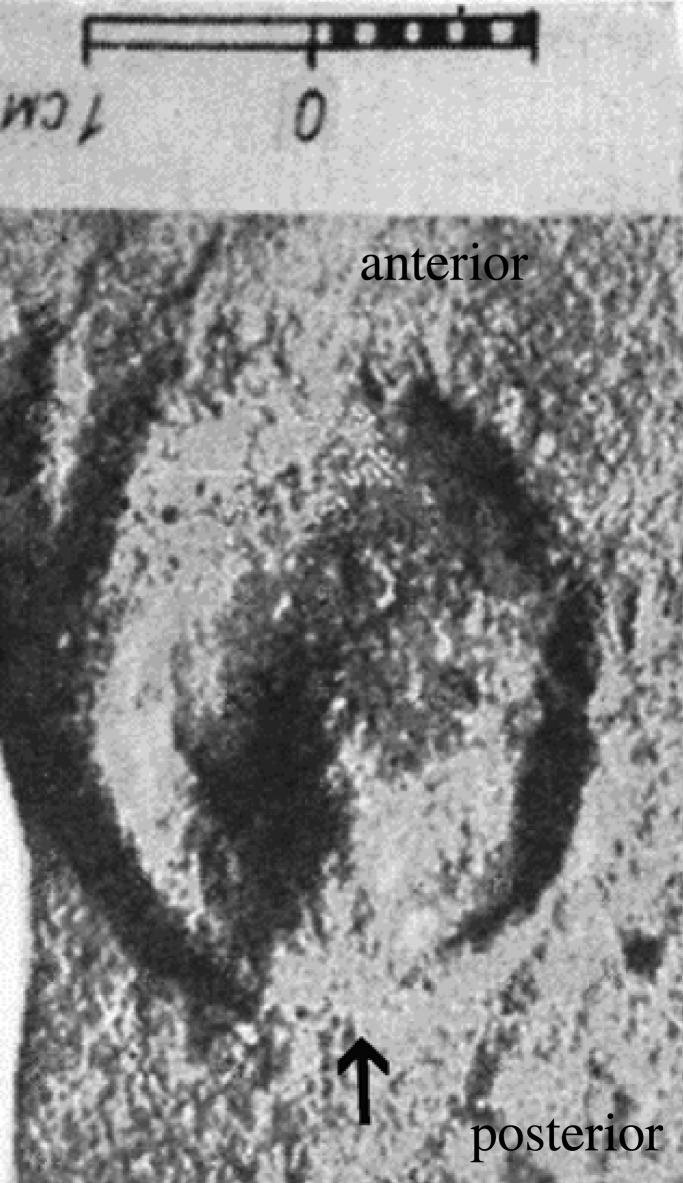
Late middle Miocene hoof impression (*Hippipeda parva*) from Bila Tserkva, Ukraine with a detailed frog visible posteriorly (indicated by arrow). Bar is 1 cm (from [102], plate II, fig. 3).

There are also ichnological indications of a frog in the footprints of some tridactyl Miocene and Pliocene equids. Some of these are often rather equivocal and open to interpretation and are detailed further in the electronic supplementary material. But a clear frog can be seen in a hoof print from the late middle Miocene outside of Bila Tserkva, Ukraine (Teresva Formation, approximately 13 Ma). This small—2.8 cm long by 2.2 cm wide—hoof print (*Hippipeda parva*), formed in marine sand, bears the clear trace of a well-developed frog at the back of the hoof [102], p. 73, tab. II, fig. 3); figure 7. It has been attributed to *Anchitherium*, which matches well with its size and geological dating, but could also be from a hipparionin equid (albeit at a relatively early date for the Old World, as hipparionin fossils are not known until approximately 11.5 Ma), as anchitheres are considered to have had a footpad. Whatever the identity of the maker of this trackway, it shows an indisputable frog in a tridactyl equid, as monodactyl equids were unknown in the Old World until the Pleistocene.

**Figure 8.**
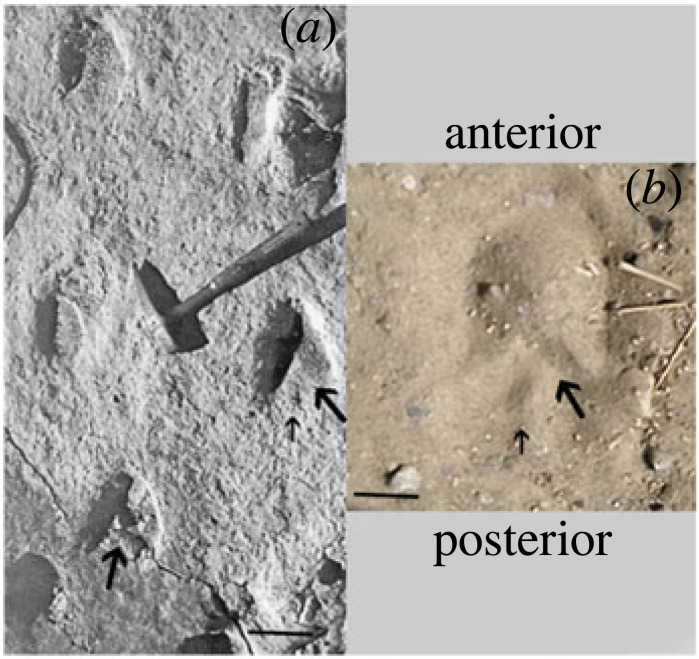
(*a*) *Hipparion* or *Cremohipparion* footprints from the late Miocene Hoya de la Sima site (Jumilla, Spain) with a distinct V-shape at the back of some of the impressions (from [103], p. 262, fig. 7). There is also an occasional indentation posterior to the V-bar, resembling that formed by the central sulcus of the frog of a modern equine. Large arrows indicate the posterior V-shape and the small arrow traces the central sulcus of the frog; (*b*) print of modern Sicilian Donkey trotting in sand with a similar V-shaped in the back (indicated by large arrow) and indentation made by the central sulcus of the frog (indicated by small arrow). Bars are 5 cm.

In addition, a V-shape (uve) occurs at the rear of footprints made by a tridactyl equid at the late Miocene (Messinian) Hoya de la Sima site mentioned above ([103], p. 262–264); figure 8*a*. The hoof prints were probably made by *Hipparion* or *Cremohipparion*, but certainly by a tridactyl hipparionin equid, as *Equus* did not enter the Old World until the start of the Pleistocene. While the frog itself is not visible in the Hoya de la Sima prints, the sharp V-shape at the posterior part of the print resembles that left by the V-bars of the posterior hoof wall and the collateral sulci in modern equines (figure 8*b*). Moreover, a few of the Hoya de la Sima prints display a small indentation running parallel to the direction of travel posterior to the V-bars that greatly resembles those formed by the central sulcus of the frog in modern equine prints (figure 8*b*), further suggesting the presence of a frog. Other trackways of probable tridactyl equids evidencing frogs are further discussed in the electronic supplementary material.

While we can have utmost certainty that the middle Miocene Ukrainian footprint was made by a tridactyl equid, in the later Miocene and Pliocene of North America both tridactyl and monodactyl equids might be present in the same deposits: how could their footprints be distinguished if no side toe imprints were present? It has been postulated that such hoof prints can be distinguished by their shape, more oval in tridactyl equids and more circular in monodactyl equids [73]. By comparing footprints and ungual phalanges of fossil and modern equids, we found that this generalization holds up quite well. Tridactyl equids tend to have ungual phalanges and footprints that are oval in shape and longer than wide (as in the Ukrainian print with frogs noted above where length/width ratio = 1.27), whereas monodactyl equids tend to have ungual phalanges and footprints that are more circular in shape and wider than long (see later discussion of the ‘ovality index’; see also [104], p. 17, fig. 10). In the electronic supplementary material, we report on other prints with frogs or V-bars that are probably tridactyl due to their being quite a bit longer than wide.

There is thus good evidence of frogs in the footprints of tridactyl equids, meaning that the *Equus* frog cannot have been formed from the distal portions of the side digits still present in those equid taxa. When the frog first evolved is not entirely clear. As a frog is evidenced in the footprints in hipparionin and perhaps protohippin taxa (see electronic supplementary material), then it must be a feature of all of the family Equinae, at least the ones more derived than the basal species of ‘*Merychippus*’: Hipparionini plus Protohippini diverged from Equini in the late early Miocene approximately 17.5 Ma [105]. It seems probable that the frog would be a feature of the hooves of all spring-footed horses and thus also present in the stem-equine Anchitheriinae *Archaeohippus*, *Desmatippus* and *Parahippus*, which first appeared in the latest Oligocene (see [4]). The intriguing possibility that the Ukrainian footprint *Hippipeda parva* actually belongs to *Anchitherium* would mean that the traditional interpretation of earlier anchitheres retaining a footpad is incorrect. Anchitheres in general differ from hyracotheres in having side digits that are considerable shorter than the central one (although not as short as in the spring-footed taxa: figure 1), unlike the condition in extant pad-footed ungulates such as tapirs, rhinos and hippos; this issue deserves further consideration, but is outside the purview of this contribution.

### 4.7. Hoof shape and the index of ovality

**Figure 9.**
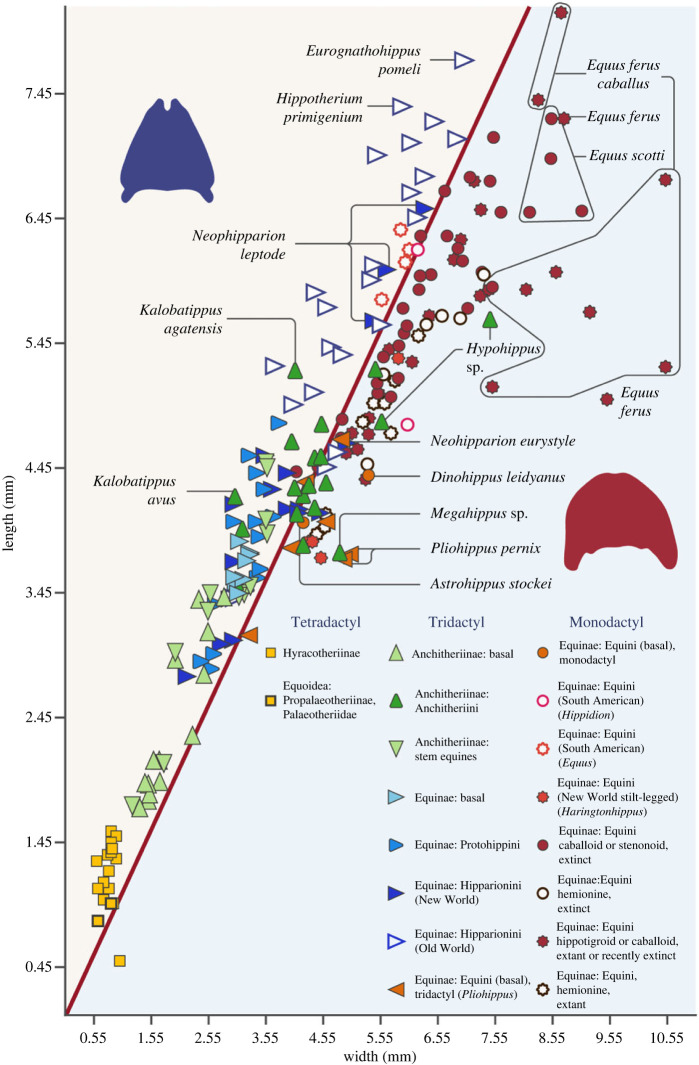
Bivariate plot of the length versus the width of the ungual phalanx of the third digit in fossil and living equids. The line drawn is one of isometry. Tetradactyl (squares) and tridactyl equids (triangles) tend to place above the line (phalanx longer than wide) while monodactyl equids (circles) tend to place below the line (phalanx wider than long). Species of the genus *Acritohippus* are included with *Merychippus* in the category ‘basal Equinae’, following [106]. *Efc* = *Equus ferus caballus*, i.e. the domestic horse. The blue ungual phalanx silhouette (plantar view), illustrating a more oval-shaped foot, is based on *Neohipparion coloradense*, F:AM 436–21757; the red ungual phalanx silhouette (plantar view), illustrating a more round-shaped foot, *Equus ferus przewalski,* AMNH 35859: both from photos taken by C.M.J. The data used to construct this plot can be found in the electronic supplementary material, table S1. Some key taxa are labelled, and electronic supplementary material, figures S13–S16 show the identity of all the taxa in the plot via numbers listed in the electronic supplementary material, table S1.

**Figure 10.**
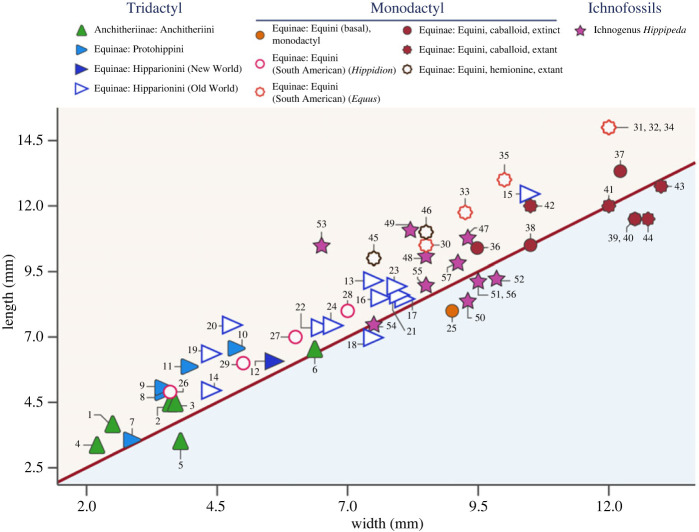
Bivariate plot of the length versus the width of footprints of fossil and extant equids. For description see figure 9. The numbers refer to the taxa listed in the electronic supplementary material, table S2. The following taxa show these additional features. A possible posterior ‘V’ or ‘U’ shape: 1 (?), 5–6 (?), 8–11, 13, 16–18. A definitive frog: 4, 34, 36–37, 38 (?), 53. V-bars; 47–48. Lateral digits: 13 (behind), 14 (to the side and behind), 16–18 (behind), 19 (behind?), 23–24 (to the side). The data used to construct this plot, along with the numbers identifying the taxa, can be found in the electronic supplementary material, table S2.6.

Figures 9 and 10 show novel data on the shape of horse feet and hoof prints: note that these figures show absolute measurements, so in general the larger taxa are the ones furthest along the line of isometry (but see comments about large anchitheres below). Figure 9 shows the shape of the ungual phalanx for a large diversity of fossil and extant taxa. However, note that fore hooves and phalanges tend to be more rounded than hind ones in *Equus* and many other species in the Equinae, ([12,73]; see electronic supplementary material, table S1): we have not indicated this difference on figure 9, but see discussion below. Basal equids (tetradactyl Hyracotheriinae and tridactyl basal Anchitheriinae) have phalanges that are slightly longer than wide. Most tridactyl Equinae have phalanges that are considerably longer than wide: i.e. more oval in shape, as well as somewhat pointed. This is especially the case for most basal equine taxa, and taxa in the Equinae tribes Protohippini and Hipparionini. By contrast, with the trend towards monodactyly in the Equini, those equids have phalanges that are wider than long: i.e. more circular in shape. This is especially true for large, extant species of *Equus*, although hemionines, i.e. asses and their relatives, tend to have more oval feet (plus they are often smaller than caballoid [horses] and hippotigrine [zebras] equids). Domestic horses (*Equus ferus caballus*) have very variable phalanx size and comprise the data points that diverge the most from the isometric line that in the main separates the tridactyl species from the monodactyl ones (figure 9). Very wide phalanges often belong to larger equid individuals, and/or to larger breeds (e.g. draft horses rather than thoroughbreds). The extinct caballoid *E. scotti* also had more rounded ungual phalanges than most other equids (figure 9).

The contrast in ungual phalanx shape is borne out by statistical analysis comparing the OI values (maximum length/maximum width) for the ungual phalanx III of tridactyl versus monodactyl equid taxa (see electronic supplementary material, table S3). All the analyses yielded a statistically significant two-sample Welch's *t*-test, *p* < 0.00001. In comparing all equids, all the taxa that have more than a single toe (i.e. hyracotheriines, anchitheriines and the majority of equines, *n* = 127) have a mean OI of 1.19 while the monodactyl equines have a mean OI of 0.88 (*n* = 96). With the removal of the tetradactyl (at least in the forefoot) hyracotheres, which also tend to have very narrow ungual phalanges, the tridactyl anchitheriines or equines (*n* = 110) have a mean OI of 1.15 for the ungual phalanx III. Restricting the tridactyl forms further to just the equines (*n* = 65), so spring-footed forms are being compared with each other, their mean OI is still 1.15. A final analysis compared this same grouping, but without the individuals of the domestic horse, *Equus ferus caballaus*, as this species can have highly variable ungual phalanges, some of them being very wide (figure 9), and it is possible that these very wide-footed individuals were biasing the monodactyl average. With these individuals removed, the mean OI for monodactyl forms (*n* = 78) was only slightly greater (0.91) and the difference between tridacyl and monodactyl equines was still highly statistically significant (see electronic supplementary material, table S3).

The hind ungual phalanges of digit III do tend to be slightly narrower and longer than those of the front from the same individual in many equids, and so overall have a slightly larger OI value (1:1.03 ± 0.11; see electronic supplementary material, table S1). However, a statistically significant large difference in mean OI between equids still occurs when comparing forefoot ungual phalanges III alone (tridactyl, *n* = 18, mean OI = 1.12; monodactyl, *n* = 17, mean OI = 0.85; *p* = 0.000001 in a Welch's *t*-test comparing these two groups) or hind foot ungual phalanges III alone (tridactyl, *n* = 18, mean OI = 1.17; monodactyl, *n* = 17, mean OI = 0.86; *p* < 0.000001) of the same individuals, or in combination (tridactyl, *n* = 36, mean OI = 1.15; monodactyl, *n* = 34, mean OI = 0.85; *p* < 0.000001).

Figure 10 shows a much smaller amount of data on hoof prints: 24 tridactyl forms and 20 monodactyl ones (no hyracotheres were included). In general, the hoof prints tend to be more oval than the underlying ungual phalanges; only a few specimens of modern horses have hoof prints that are wider than long. However, the overall difference in shape between tridactyl and monodactyl equids remains, with monodactyl forms having rounder hooves (see also electronic supplementary material, tables S1 and S2).

A statistical analysis of the OI of the hoof prints of all tridactyl forms (*n* = 24, mean OI = 1.22) compared with all monodactyl forms (*n* = 22, mean OI 1.14) does not show a significant difference (see electronic supplementary material, table S3). However, if the heminones and the South American monodactyl (sub)genera of *Hippidion* and *Amerhippus* are excluded, both of which tend to have hooves that are longer than wide, then a statistically significant result is obtained (*p* = 0.00007; see electronic supplementary material, table S3) when performing a two-sample Welch's *t*-test comparing the OI of tridactyl hoof prints with the remaining monodactyl forms (*n* = 10, mean OI 0.99).

Equids became monodactyl and lost their side toes some time prior to the evolution of *Dinohippus* at a body mass of approximately 200 kg (see [35,107]). At this point, a wider hoof would have increased traction and stability, and a wider hoof also would have distributed the weight load over a larger surface area reducing underfoot pressures and therefore sinking depth along with chance of injury [108]. Note (as shown in figure 9) that among the basal equins, the possibly tridactyl *Astrohippus* has more oval phalanges than the likely monodactyl *Dinohippus*. Individuals of *Pliohoppus* tend to plot close to the isometric line, but two individuals of *P. pernix* have more rounded phalanges; it would be interesting to know if these individuals were monodactyl ones, as side digit size is variable in individuals of this species [32]. A larger and broader foot would help reduce underfoot pressures, especially in larger animals, and may be essential for weight bearing in a monodactyl taxon. Such large feet would offer greater stability over softer substrates and reduce stress on firmer substrates. Note though that although there were a number of hipparionin species, especially in the Old World, that exceeded 200 kg [109], these did not develop as broad or rounded a hoof as in monodactyl taxa (figure 9), possibly because of the support of the side digits.

Exceptions to this pattern of phalanx evolution include the large, derived (usually greater than 200 kg) middle to late Miocene anchitheriin genera *Hypohippus* and *Megahippus*, which have notably rounded ungual phalanges (figure 9), and possibly also rounded hoof prints (figure 10). Some of these anchitheriins were of similar size to modern *Equus* species, or the large Old World hipparionines like *Eurygnathohippus*, but their third digit ungual phalanges as plotted on figure 9 are absolutely smaller than these other large equids (see also figure 3). Large anchitheriins differed from monodactyl equins in their tridactyl and probably padded foot, although they resembled them in having a broad fetlock joint: they may have possessed a unique type of foot stabilization, perhaps an adaptation for life in more closed habitats (see [30]). Some large New World hipparionins such as *Neohipparion* also generally show ungual phalanges and footprints that are more circular that those of other hipparionine taxa (see figures 9 and 10).

In addition, the monodactyl South American Equini genera *Amerhippus* and *Hippidion*, as well as some extinct hemione-like *Equus* specimens (sometimes classified as *E*. ‘*conversidens*’, or alternatively placed in a new genus *Haringtonhippus* (see figures 9 and 10, table 1, and electronic supplementary material, tables S1 and S2, and figure S15)), differed from other monodactyl horses in having ungual phalanges and footprints somewhat longer than wide (perhaps an adaptation to life on harder and drier lands or in more mountainous regions; see [96,108,112–114]). Note that tridactyl equids were never present in South America, so we can be certain that these prints were made by monodactyl taxa.

**Table 1.** OI (length/width) for equid phalanges and hoof prints.

equid group	ovality (length/width) of ungual phalanges III	ovality (length/width) of hoof prints
Hyracotheriinae	1.51 (0.50–2.17)	—
Anchitheriinae (basal)	1.23 (1.00–1.46)	—
Anchitheriinae (stem Equinae)	1.25 (1.08–1.53)	—
Anchitheriini	1.02 (0.75–1.39)	1.15 (0.79–1.44)
Equinae (basal) (= *Merychippus sensu stricto* following [105]; and *Acritohippus* following [106])	1.17 (1.09–1.35)	—
Protohippini	1.19 (1.06–1.40)	1.38 (1.10–1.50)
Hipparionini (New World)	1.13 (0.92–1.40)	1.11 (1.11)
Hipparionini (Old World)	1.14 (0.96–1.43)	1.19 (0.95–1.58)
Equini (basal) = genera *Pliohippus, Astrohippus* and *Dinohippus*	0.92 (0.76–1.10)	0.89 (0.89)
Equini (South American) = genera *Hippidion* and A*merhippus*	0.99 (0.75–1.08)	1.24 (1.14–1.36)
Equini (New World stilt legged, *Harringtonhippus* following [110,111])	0.88 (0.83–0.91)	—
*Equus* Caballoid (extinct)	0.91 (0.72–1.08)	1.06 (1.00–1.10)
*Equus* Stenonoid (extinct)	0.94 (0.88–1.01)	—
*Equus* Hemione (extinct)	0.86 (0.82–0.93)	—
*Equus ferus* Caballoid (extant and recently extinct)	0.77 (0.50–0.94)	0.98 (0.90–1.14)
*Equus* Hemione (extant and recently extinct)	0.88 (0.83–0.92)	1.31 (1.29–1.33)
*Equus* Hippotigroid (extant and recently extinct)	0.90 (0.83–0.95)	—

## 5. Developmental and anatomical evidence concerning distal side digit remnants in *Equus*

### 5.1. Developmental evidence

The embryological evidence and the anatomy of the equid pedal nerves and blood vessels do not support the hypothesis of [18] regarding the retention of distal remnants of the side digits in modern horses.

In examining the developmental evidence, it is critical to identify the stage of embryonic/fetal development. Kavanagh *et al*. [34] examined horse embryos at approximately 31 to 35 days post-conception, at the point where differentiation of the limb buds is beginning. At that point, they note that there are no condensations of mesenchymal tissue in the position of the distal segments of any digit other that the third, although there are identifiable condensations marking the position of the proximal portions of all five digits. That is, there is no evidence for the retention of any distal portions of digits I, II or IV, V. It has also been noted [115] (see also [116]) that the distal-most portions of metapodials II and IV condensations undergo apoptosis, so that only the proximal parts remain. Franciolli *et al*. [117] examined equine fetuses between 15 and 107 days post-conception and note that the pelvic and thoracic limbs are completely formed by day 34 and that the formation of the hoof starts at approximately day 27 (see their fig. 1*a*,*b*). The limbs are completely formed by day 107. Although they do not specifically address the issue of side digits, they do not mention any evidence of these in the specimens they examined. Barreto *et al*. [118] examined development in equine specimens ranging from 21 to 105 days post-conception, and described the hoof at days 45 and 55 in considerable detail. Their fig. 3*a* illustrates a cross-section through the hoof at 45 days, clearly showing the corneal tissue of the walls, sole and the region where the frog will develop. By day 55, the hoof is well developed. Again, there is no evidence of any distal segment of the side digits.

The equine embryos examined by Solounias *et al*. [18] were at a considerably later stage in development; they estimated their age at 185 and 193 days post-conception. They described ‘four folds’ in the wall (outer capsule) of the hoof, on the ventral surface, which they believed represented digits I, II, IV and V. This interpretation seems unlikely since the wall of the hoof capsule, including the sole and frog, forms considerably earlier in development, and a multi-partite structure is not described in any of the studies previously cited. Nor is there any evidence of those digits earlier in development in the studies cited above, making it unlikely that they would reappear at a later stage of development.

An additional difficulty with their interpretation of the ‘folds’ is that Solounias *et al*. [18] examined the deciduous capsule of the hoof. Early in hoof development, the wall of the hoof lacks the papillary ridge structure and tubular keratin of more mature hooves; these structures develop later in fetal development and post-natally [78,92,119,120]. Bragulla & Homberger [93] examined the timing of formation of the papillary ridge structure and the processes of soft and hard keratinization during development of the equine fetus, and demonstrated that all parts of the fetal hoof show the presence of a stratum granulosum at some stages of development. The stratum granulosum is associated with the production of profilaggrin keratohyaline granules, which is in turn related to water retention and soft keratinization. All parts of the fetal hoof produce softer keratin early in development, with this stage lasting later in development in the sole and frog, and permanently in the bulbs of the heel and coronet. Thus, the fetal/new-born hoof is soft and unpigmented [12,121,122].

### 5.2. The fetal equine ‘claw’

It has been claimed that horses are born with a claw on the front of their hooves [18,123] that is retained into the adult in the tridactyl equid *Hipparion* [18]. Where does this claw come from, is there really a definitive claw in neonatal modern horses, and is there any evidence for the retention of a claw in adult tridactyl equids?

During later stages of fetal development, as the hoof wall, sole and frog continue to grow and begin to produce hard keratin, the soft keratin is displaced distally, where it forms a soft mass covering the ends and plantar surface of the hoof by the time of birth [12,121,122]. This soft covering (eponychium), commonly referred to as ‘foal slippers’, is thought to prevent injury to the fetal membranes and birth canal by the harder mature keratin of the hoof wall beneath it [12,121,122]. It is rapidly worn off as the foal begins to walk [117]. Similar structures have been described in neonate calves, sheep and also piglets, where the side digits are still present [93,121,122,124,125]. These ‘foal slippers’ appear to have been conflated by [18] with the ‘claw’ they picture ([18], fig. 8*d*), as it is stated that the ‘claw’ is not worn off until sometime after birth [18,123].

These soft keratinous structures in foals appear to be highly variable and are generally described as leaf-like masses. The hooked structure observed by [18] and interpreted as a ‘claw’ is present at an earlier stage of development (estimated gestational time 180–195 days), not in neonates (full gestation period is approximately 330 days), and it is not present in all specimens. Illustrations in other studies show that the fetal hoof tends to be more pointed and tulip-shaped than a mature hoof, and that tip may show a somewhat hooked shape [77,93,117,118], but not the cat-like clawed structure illustrated by [18]. We consider the pointed shape of the hoof in equine fetal development to result from earlier growth from the coronary region, which adds length to the hoof, especially at the tip, while later development of the parietal papillary organ, which produces the inner layer of horn and increases circumference [77], ultimately results in the ‘bell-shaped’ hoof of adult horses. Additionally, because the soft keratin deciduous hoof capsule is highly hydrated, we believe that little can be deduced from its exact shape when it is removed from amniotic fluid, as any drying would distort its shape.

Thus, we do not consider it appropriate to refer to this structure as a ‘claw’; not only is it part of a variable soft keratin deciduous structure, but it also lacks the characteristics of a true mammalian claw, and there is no mechanism to maintain this shape in the equine hoof after birth. Mammalian claws, like hooves, consist of a hard keratin sheath, which takes its shape from the underlying dermal layer, which in turn follows the contours of the supporting ungual phalanx and is attached to the periosteum [77,125–127]. The keratinous sheath may differ somewhat in its exact outlines depending on the thickness of the keratin in different parts of the claw, the structure of the keratin and environmental factors, such as wear [128–130]. Still, the ungual phalanx of clawed mammals, as well as reptiles and birds, has an anterior hooked structure which defines the shape of the claw [128–130], although some of the mechanisms of keratinization may differ [131]. The ungual phalanx in living equids clearly does not have such a hooked protuberance to support the shape of a claw, but instead is round in shape, with a small notch (crena marginalis) matching that of the rounded hoof [132]. In *Hipparion*, the ungual phalanx is somewhat less rounded anteriorly and has a small notch (cleft or fissure) on the anterior border that Solounias *et al*. [18] believe supports an adult claw. It would not, however, be mechanically feasible for such a notch to support the protruding underlying dermal and germinal layers that would be necessary to produce a solid hooked claw. A similar notch is seen in the ungual phalanges of some other early perissodactyls, a few condylarths (small- to medium-sized Palaeogene archaic ungulates), and the monodactyl litoptern *Thoatherium*, although we do not know of any instances in extant taxa, and its function is unknown. However, chalicotheres (large fossil perissodactyls) have fissured ungual phalanges that do appear to have supported claws, but the entire phalanx is elongated and arched [133]. The fissure might have been reflected in a groove on the surface of a claw that followed the contour of the phalanx, but a notch or fissure alone would not support a keratinous claw or claw-like structure. The loss or reduction of this cleft may simply be the result of change in ungual phalanx shape in monodactyl equids, as earlier discussed, and it is exceedingly unlikely that any equid had a ‘claw’ that existed beyond early fetal development.

### 5.3. Origin of the frog and the hoof collateral cartilages

As described above in §4.4, the frog is a specialization of the anterior projection of the bulbs onto the plantar surface of the posterior hoof (heel); the heel bulbs project anteriorly into the sole in a wide range of ungulates. Only the degree of specialization in equids is unique, suggesting that there is no need to invoke incorporation of additional digits to explain its evolutionary origin. Bragulla [77] examined the development of the papillary organ in the entire hoof capsule and noted that at less than 60 days gestational age, when differentiation of the hoof tissues begins, the continuous epidermis of the hoof capsule rests on a continuous basement membrane separating it from the undifferentiated underlying dermis; that is, there are no subdivisions indicating origin from separate sources. This study traces the development of the dermal papillary organ in this continuous dermal layer through neonatal stages and does not report any divisions that might indicate incorporation of digits II or IV into the hoof capsule; all differences in the differentiating dermal papillary organ reflect the functional differences in that organ and in adults. Since no studies of early embryonic development reported the presence of distal segments of digits I, II, IV or V that might have been available to be incorporated into the hoof capsule of digit III [34,117,118], there is no evidence to support any contribution to the frog from digits II or IV.

Additional evidence that only digit III contributes to the frog is provided by some so-called polydactyl horses (variants of regular domestic horses) that possess at least one additional digit (see [33,134–137]). Although such polydactyly has different developmental causes and is not necessarily a reversion to an ancestral state, i.e. atavism (see [138]), in some cases, the supernumerary digit has its own carpal articulation and innervation and bears a resemblance to the ancestral condition. In at least one described case, the hoof on digit III is normal despite the presence of accessory digits and the supernumerary digits also have a frog [139]. This is quite contradictory to the notion that the frog has been formed from retained distal portions of the side digits.

There is also no developmental evidence to support the hypothesis that the plantar processes and collateral hoof cartilages (hoof wings) are derived from digits I and V, since the distal portions of those digits are not reported in any developmental study [34,117,118]. Although plantar processes are absent in the tetradactyl manus of hyracotheres, they do occur on each side of the ungual phalanx of digit III of the four-toed manus of tapirs ([8], p. 3; figure 1), with the implication that the formation of these processes is independent of the incorporation of other digits.

### 5.4. Arterial and nervous supply of the foot

Solounias *et al*. [18] considered that the presence of multiple artery/nerve bundles in cross-sections of the equine embryos they examined were proof of the retention of digits I, II, IV and V and their arterial supply. If this were true, it would be expected that these vessels were not only present, but also that they would supply the structures believed to be derived from these digits, namely the frog and the digital cartilages. However, this is not the case. The common mammalian pattern of arterial supply to the single digit is maintained in horses, with a single lateral and medial vessel on the palmar and plantar surfaces (from here on simply referred to as ‘plantar’); these are the medial plantar and lateral plantar digital arteries [67,76]. These arteries supply the hoof and all its structures. More proximally, the plantar metacarpal (metatarsal) arteries of digits II, III (medial palmar or dorsal plantar artery), IV and V are present, but only the medial palmar (dorsal plantar) artery branches to form the lateral and medial plantar digital arteries supplying the foot. The other plantar metapodial arteries terminate in anastomoses with the lateral or medial plantar arteries [67,76].

The plantar digital arteries give off numerous branches supplying the phalanges and interphalangeal joints [67,76]. We believe it is these branches that were interpreted as continuations of digital arteries [18]. The hoof itself is highly vascularized to support the complex papillary body in the wall, sole and frog [67,76]. The frog and collateral cartilages are not separately supplied, as would be expected if they were in fact derived from digits I, II, III and IV. Similarly, the innervation of the foot follows the common mammalian pattern, with the medial plantar nerve proper and the lateral plantar nerve proper supplying the medial and lateral sides of each digit; each of these nerves also provides a dorsal branch [76]. There is no evidence for separate innervation of the frog and collateral cartilages.

## 6. Conclusion

Solounias *et al*. [18] present the novel idea that remnants of each of the five digits of the mammalian pentadactyl limb, retained to a certain extent in ancient equids, are found in adult present-day monodactyl horses. They argue that proximal remains of metacarpals I and V form proximal ridges on the splint bones (i.e. metacarpals II and IV), which are themselves attached to the side of the cannon bone (i.e. metacarpal III). This idea is confirmed by the developmental evidence provided in Kavanagh *et al*. [34]. However, their contention that distal remains of digits II and IV intersect to form the V-shaped frog on the posterior portion of the hoof, and that distal remains of digits I and V form the plantar processes plus collateral cartilages of the ungual phalanx, is shown here to lack support.

Frogs are not always apparent in modern horse footprints, and so their absence in a fossil print does not mean an absence in foot anatomy. Moreover, there is good ichnological evidence that some tridactyl equids did indeed possess frogs. Fossil equid trackway evidence does not then support the notion that the frog of modern horses is formed from the distal portions of digits II and IV: if this were true then no footprint of a tridactyl equid would show a frog. The notion that *Hipparion* possessed a footpad is also refuted by the osteology of the foot of derived tridactyl equids (i.e. derived Anchitheriinae plus hipparionin and protohippin Equinae) which clearly shows an *Equus*-like spring foot with an unguligrade foot posture and loss of the footpad (see [17]).

Further confirmation of the frog being formed independently of any distal remnants of side digits is found in the occasional modern horse born with an additional vestigial toe or toes, so-called polydactyl horses [134]. In some situations of polydactyly, it appears that a frog is present in both the central toe and the side toes [139], contra-indicative to the frog of the central digit being formed from elements of side toes.

While the developmental studies of Kavanagh *et al*. [34] and other researchers show evidence for the retention in modern horses of the proximal portion of all of the original pentadactyl digits, they show no evidence for the retention of distal portions of the additional digits in ontogeny. Such retention would be a necessary condition of the proposal of [18] that distal portions of digits II and IV contribute to the frog and digits I and V to the ‘hoof wings’. The frog appears to have arisen independent of any co-option of the remains of the distal side toes, and the illustrated ‘hoof wing’ represents a pathological condition in the ungual phalanx of an older domestic horse.

The composition of the hoof of modern horses was probably also the form of the hoof of the central toe (digit III) in many of the extinct tridactyl forms. The frog that characterizes the sole of the *Equus* foot can be shown to be present in fossil hoof prints of tridactyl equids. Small frogs may also have been present on the hooves of the side digits of tridactyl equids, as sometimes seen today in the accessory digits of polydactyl domestic horses. The original equid footpad was certainly lost in the spring-footed forms (Equinae and derived Anchitheriinae) and may have been lost in more basal anchitherines, as suggested at least by one fossil footprint. The equid frog and bulbs of the heel may well represent a remnant of the original footpad, retained in a modified form as a shock-absorbing structure and has probably been a feature of the hoof for much of equid evolutionary history. The feet of modern horses may technically be polydactyl proximally, but they are definitely monodactyl distally.

## Museum abbreviations

AM or AMNH = American Museum of Natural History, New York, USA. F:AM = Frick collection, American Museum of Natural History. USNM = United States National Museum, Washington, USA. MCZ = Museum of Comparative Zoology, Harvard University, USA.

## Data Availability

All data are provided within the main text and figures of this article with the exception of the data for figures 9 and 10. The data used to generate these figures are given in the electronic supplementary material [140], tables S1 and S2, representing the specimens measured, the measurements of equid bones and footprints, and the published sources or museums from which the measurements were derived. The tables are followed by the relevant museum abbreviations as well as full source information with DOIs and linked URLs. Such raw factual data (measurements of observable objects in the world) used in our study are not copyright protectable and subject to fair use in open access publications where reference is given to source of data, and indeed our particular use is transformative in generating a new chart from the data.
